# Toward dissecting the etiology of schizophrenia: HDAC1 and DAXX regulate GAD_67_ expression in an *in vitro* hippocampal GABA neuron model

**DOI:** 10.1038/tp.2015.224

**Published:** 2016-01-26

**Authors:** S Subburaju, A J Coleman, W B Ruzicka, F M Benes

**Affiliations:** 1 Program in Structural and Molecular Neuroscience, McLean Hospital, Belmont, MA, USA; 2 Department of Psychiatry, Harvard Medical School, Boston, MA, USA; 3 Program in Neuroscience, Harvard Medical School, Boston, MA, USA

## Abstract

Schizophrenia (SZ) is associated with GABA neuron dysfunction in the hippocampus, particularly the stratum oriens of sector CA3/2. A gene expression profile analysis of human postmortem hippocampal tissue followed by a network association analysis had shown a number of genes differentially regulated in SZ, including the epigenetic factors HDAC1 and DAXX. To characterize the contribution of these factors to the developmental perturbation hypothesized to underlie SZ, lentiviral vectors carrying short hairpin RNA interference (shRNAi) for HDAC1 and DAXX were used. In the hippocampal GABA neuron culture model, HiB5, transduction with HDAC1 shRNAi showed a 40% inhibition of HDAC1 mRNA and a 60% inhibition of HDAC1 protein. GAD_67_, a enzyme associated with GABA synthesis, was increased twofold (mRNA); the protein showed a 35% increase. The expression of DAXX, a co-repressor of HDAC1, was not influenced by HDAC1 inhibition. Transduction of HiB5 cells with DAXX shRNAi resulted in a 30% inhibition of DAXX mRNA that translated into a 90% inhibition of DAXX protein. GAD1 mRNA was upregulated fourfold, while its protein increased by ~30%. HDAC1 expression was not altered by inhibition of DAXX. However, a physical interaction between HDAC1 and DAXX was demonstrated by co-immunoprecipitation. Inhibition of HDAC1 or DAXX increased expression of egr-1, transcription factor that had previously been shown to regulate the GAD_67_ promoter. Our *in vitro* results point to a key role of both HDAC1 and DAXX in the regulation of GAD_67_ in GABAergic HiB5 cells, strongly suggesting that these epigenetic/transcription factors contribute to mechanisms underlying GABA cell dysfunction in SZ.

## Introduction

Schizophrenia (SZ) is a debilitating mental disorder, which affects approximately 1% of the world population and is responsible for about 3% of the disease-related economic burden.^1^ SZ is characterized by a complex interaction between genetic predisposition and environmental influences that may lead to disturbances of early brain development.^2, 3^ GABA neurons in the cortex and hippocampus of SZ are subject to pathophysiological changes whose key feature is a decrease in glutamate decarboxylase (GAD) mRNA^4, 5^ and -immunoreactivity.^6^ The resulting loss of GABAergic activity disrupts cortical gamma oscillations and may account for the cognitive deficits seen in SZ.^7^ Understanding the molecular mechanisms of GABA dysfunction in SZ is critical for the development of effective new treatments for the disease. Using a combination of laser microdissection and gene expression analysis of human postmortem hippocampal tissue, a previous network association analysis detected a number of genes associated with the regulation of GAD_67_ expression that were abnormally regulated in SZ compared with normal controls.^8, 9, 10^ This abnormal expression of genes within the network was specifically found in the hippocampal stratum oriens (SO) of sector CA3/2. Although some of these genes had previously not been linked to SZ, an analysis of copy number variation (CNV) and gene expression extended and confirmed these differences for some genes previously identified by network association analysis.^11^ Two of the genes (that is, HDAC1 and DAXX) are co-repressors associated with epigenetic regulation that help to control promoter histone acetylation reactions involved in regulating GAD_67._^12, 13, 14, 15^

HDAC1 serves as a repressor of promoters, including that of the GAD1 gene, by forming complexes with a number of other epigenetic- and transcription factors.^16, 17^ HDAC1 expression and gene copy number are increased in the brain of SZ compared with controls.^8, 11^ DAXX, a transducer of growth-regulatory signals and a transcriptional and epigenetic regulator, also acts as a co-repressor of promoters through an association with HDAC1. ^8,13^ Both HDAC1 and DAXX are upregulated at a principle locus where GAD_67_ expression is decreased that is, in the hippocampus of SZs.^8^ This increase of HDAC1 and DAXX was observed only in CA3/2 of SO, but not in adjoining hippocampal regions. In bipolar disorder (BD), HDAC1 and DAXX expression were not affected at the same locus of the hippocampus, despite the fact that GABA neuron dysfunction is also present in this region of the hippocampus in BD.^8^

Despite having limitations,^18^ postmortem studies of SZ are an important first step in understanding the molecular mechanisms of this disease.^18^ Although HDAC1 and DAXX are abnormally regulated in SZ, it has not as yet been established that changes in their expression may alter the expression of GAD_67_. To shed further light on this issue, this study has employed a novel *in vitro* model in which hippocampal precursor cells are differentiated into neurons with a GABAergic phenotype *in vitro*.^19^ Lentiviral vector (LVV)-based short-hairpin RNAs are used to selectively inhibit short hairpin RNA interference (shRNAi) the expression of HDAC1 and DAXX in cultured neurons with a GABAergic phenotype, so that their ability to regulate the expression of GAD_67_ can be investigated without potential interference from the influences of larger neural circuitry in which the GABA cell might be found. The current report explores the question as to whether HDAC1 and DAXX may play a basic role in the cell-autonomous regulation of GAD_67_ expression in GABAergic neurons.

## Materials and methods

### Cell culture

Neural precursor SV40 large T-antigen-immortalized HiB5 cells originally prepared from Sprague Dawley rat embryonic (E16) hippocampus^20^ were kindly provided by Prof. Anders Björklund, Lund, Sweden. Cells were expanded on arrival and aliquots frozen. All experiments were performed with cells which had undergone not more than three passages after re-culturing. The cultures were maintained in proliferation medium (Dulbecco's Modified Eagles Medium (Invitrogen, Grand Island, NY, USA) supplemented with 10% fetal calf serum (Gibco-BRL, Grand Island, NY, USA) in 1% Penicillin–Streptomycin) and a 5% CO_2_ environment at 33 °C. To induce and maintain differentiation, cells were incubated at 39 °C in N2-supplemented serum-free medium (DMEM/F12 1:1). After 2 days at 39 °C, platelet-derived growth factor (PDGF; 30 ng ml^−1^; Sigma, St Louis, MO, USA) was added and the cells were incubated for another 2 days. Then, 50 ng ml^−1^ BDNF was added to the media containing PDGF for an additional 2-day period. BDNF signaling through TrkB is a key regulator of GABA expression and GAD_67_ function during development.^21, 22^ Differentiated HiB5 cells show morphological characteristics of neurons, express post-mitotic neuronal markers, synthesize GABA and release it on stimulation^19^ and integrate into the developing hippocampus.^20^

### Immunocytochemistry

Differentiated HiB5 cells were fixed with ice-cold 4% paraformaldehyde in 0.1 m phosphate buffer (pH 7.4) for 20–30 min. A standard immunofluorescence technique was used to visualize epigenetic factors.^19^ Antibodies used included mouse anti-HDAC1 1:750 (Cell Signaling, Danvers, MA, USA; 5356) and rabbit anti-DAXX 1:750 (Santa Cruz, Santa Cruz, CA, USA; sc 7152).

Postmortem human hippocampus (healthy control) was cut into 25  μm sections. The immunofluorescence technique was basically the same as above for HiB5 cells. Antibodies were anti-GAD_67_ (monoclonal mouse, 1:250, Millipore, Billerica, MA, USA; MAB 5406), rabbit anti-HDAC1 (1:150, Santa Cruz; sc 7872) and rabbit anti-DAXX (1:150).

Fluorescence Alexa 488 (mouse A21202) 594 (rabbit A21207)-coupled secondary antibodies were raised in donkey (Invitrogen) and used at 1:600. Fluorescent staining was analyzed using a Leica fluorescent microscope (Wetzlar, Germany).

### Transduction of HiB5 cells with LVVs expressing shRNAi

Specific gene sequence targets were selected using the RNAi consortium (TRC-Sigma) Broad Institute algorithm. Three shRNA constructs that target distinct 21mer sequences within the HDAC1 or DAXX genes were designed and cloned into the pLKO.1 vector. The U6 promoter drives the shRNA target gene sequences and a TurboGFP reporter (St Louis, MO, USA) is under the control of a CMV promoter. All target vector constructs and the respective viral particles were custom made by Sigma-Aldrich (St Louis, MO, USA) (Table 1). An initial experiment using different amounts of lentivirus particles (for example, 1, 5, 10 lentivirus particles per cell) was performed to identify the extent of transduction and the optimal multiplicity of infection. The transduction efficiency of each target was then determined using fluorescence microscopy typically 3 days after infection. The cells were observed every day after infection to check for toxicity and/or the start of TurboGFP reporter expression. Based on transduction efficiency of lentivirus particles in HiB5 cells, an multiplicity of infection of 10 particles per cell was used in subsequent experiments and to screen for the most efficient target shRNAi sequence for HDAC1 and DAXX genes. In a pre-selection process, the most efficient shRNAis for HDAC1 (5′-AGCGACGACTACATCAAATTC-3′) and DAXX (5′-AGCTCTGCACTGTCCTTAAAG-3′) were identified.

**Figure 1:**
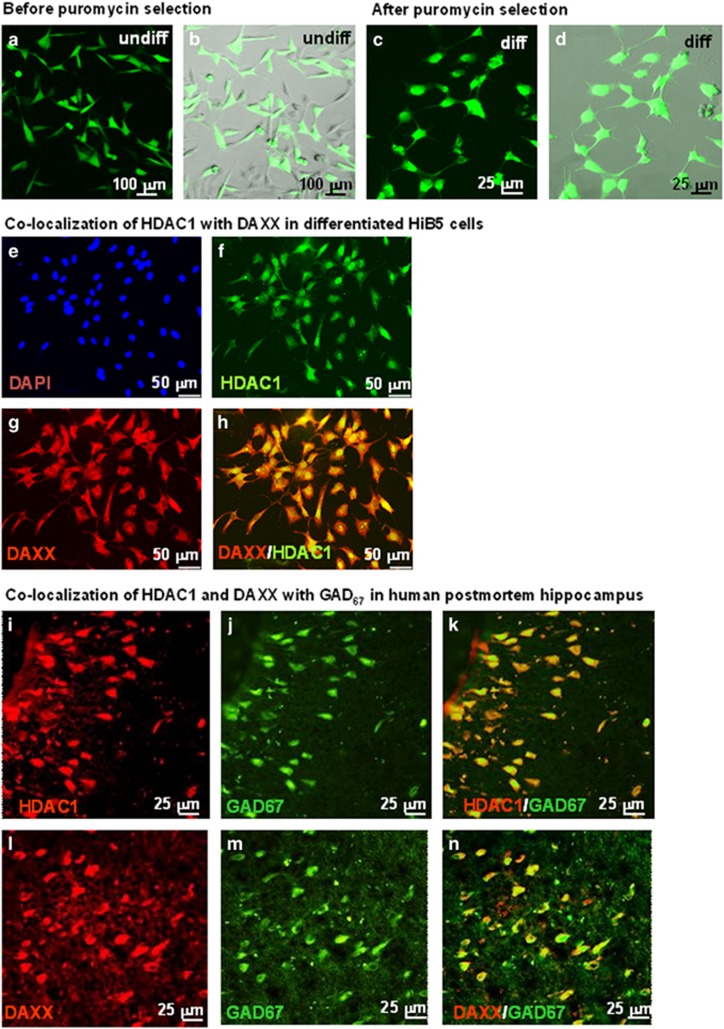
Selection of LVV-transduced HiB5 cells using puramycin and co-localization of HDAC1 and DAXX in differentiated HiB5 cells and HDAC1/GAD_67_ or DAXX/GAD_67_ in human postmortem hippocampus. For puromycin selection, differentiated HiB5 cells were transduced with lentiviral vectors. Transfected cells were incubated for 24 h and then re-plated into petri dishes for puromycin selection (2 μg ml^−1^, 1 week). Virus-infected cells express a resistance gene for puromycin and survived, non-infected cells were eliminated by the antibiotic. (**a** and **b**) Cells before treatment with puromycin; (**c** and **d**) Cells after treatment with puromycin; (**a** and **c**). tGFP fluorescence; (**b** and **d**) tGFP and phase contrast microscopy. For co-localization of HDAC1 and DAXX, differentiated HiB5 cells (*n*=3 samples) were fixed with 4% paraformaldehyde. Epigenetic factors were detected using specific antibodies, followed by fluorescence visualization. (**e**) DAPI (blue fluorescence, nuclei). (**f**) HDAC1 (green fluorescence). (**g**) DAXX (red fluorescence). (**h**) Overlay of HDAC1 and DAXX. For co-localization of HDAC1/GAD_67_ and DAXX/GAD_67_ in postmortem human hippocampal SO of sector CA3/2, 25- μm sections of human control hippocampi (*n*=3) were incubated with specific antibodies, followed by visualization using fluorescence. (**i–k**) Colocalization of HDAC1 with GAD_67_; red HDAC1, green GAD_67_, yellow overlay. (**l–n**) Colocalization of DAXX with GAD_67_; red DAXX, green GAD_67_, yellow overlay.

For transduction, proliferating HiB5 cells (20 000 cells per 50 μl) were incubated in an Eppendorf tube with lentiviral particles containing shRNAi (10 lentiviral particle per cell) for 4 h at 33 °C to maximize cell or virus contact. An empty LVV without RNAi sequence and a non-target RNA sequence-containing vector (will activate RISC and the RNAi pathway but not target any rat genes) were used as controls. Transduced cells were plated onto six-well plates, incubated for 24 h and then re-plated into petri dishes (10 cm) for puromycin selection (2 μg ml^−1^, 1 week). Virus-infected cells express a resistance gene for puromycin and survived, non-infected cells were eliminated by the antibiotic (Figures 1a and d). Surviving cells expressing the tGFP reporter were washed and switched to differentiation conditions according to the standard differentiation protocol for GABA neurons (2 days incubation at 39 °C in N2 medium; 2 additional days of incubation in N2 medium plus PDGF (30 ng ml^−1^); 2 more days of incubation with N2 medium, PDGF and BDNF (50 ng ml^−1^).

Cells were harvested, the RNA extracted using the Trizol reagent (Life Technologies, Grand Island, NY, USA), and purified using the RNeasy Lipid Tissue Mini Kit (Qiagen, Valencia, CA, USA) according to the manufacturer's instructions. The RNA was assessed using the NanoDrop 2100 spectrophotometer (Thermoscientific, Hercules, CA, USA), and an Agilent 2100 Bioanalyser (Agilent Technologies, Palo Alto, CA, USA). Alternatively, cells were harvested and the protein was extracted for western blot analysis.

### TaqMan gene expression assay-based real-time PCR

Cells were analyzed for expression of HDAC1, HDAC2, DAXX, GAD1, parvalbumin (PV), GAT1 and egr-1 mRNA using the TaqMan qPCR gene expression assay system (Life Technologies). Complementary DNA (cDNA) was generated from 1 to 2 μg RNA per sample using the High capacity cDNA synthesis kit (Applied Biosystems, Life Technologies). The expression levels of mRNA for various genes were measured by real-time PCR using TaqMan Gene Expression assays on Chromo4 Continuous Fluorescence Detection System (Bio-Rad, Hercules, CA, USA). For each sample and gene, three replicates were run in a 96-well plate. The probes contain a 6-carboxy-fluorescein phosphoramidite (FAM dye) label at the 5′ end of the gene and a minor groove binder and nonfluorescent quencher at the 3′ end and are designed to hybridize across exon junctions. β-2-microglobulin was used as an endogenous control. Real-time PCR reactions were carried out using 5 μl (1:5 diluted) of cDNA for each reaction in a 20 μl volume following the manufacturer's protocol. Gene expression values were determined as Δ*C*_t_ (*C*_t_−*C*_t β2M_) and fold changes between different samples were determined as ΔΔ*C*_t_ (Δ*C*_t vehicle_−Δ*C*_t_).

### Western blot

Cultured cells were washed with ice-cold phosphate buffered saline (PBS) and harvested in lysis buffer (T-PER Neuronal protein extraction reagent, Pierce, Grand Island, NY, USA) containing 10 μl ml^−1^ Halt protease inhibitor cocktail (Pierce). Protein extracts were stored at −80 °C. Forty micrograms of protein extract per sample were separated on 8% SDS–polyacrylamide gels and transferred onto a Hybond-PVDF membrane (GE Healthcare, Piscataway, NJ, USA). Subsequently, the blot was incubated with specific antibodies against the target genes (anti-HDAC1 (1:1000), anti-DAXX (1:500), anti-GAD_67_ (1:500; Santa Cruz; sc 5602), anti-GAPDH (1:5000; Cell Signaling; 2118 S)) overnight at 4 °C. Blots were washed in TBST and incubated with peroxidase-conjugated donkey anti-rabbit (NA934) or anti-mouse IgG (NA931) (1:10000, Amersham Biosciences, Piscataway, NJ, USA), respectively, for 30 min. Following a final wash, bands were visualized using chemiluminescence ECL prime Western Blotting detection Reagents (GE Healthcare) and the chemiluminescence signal was captured with Molecular Imager ChemiDOC XRS (Bio-Rad). For statistical evaluation, blots were analyzed using the public domain NIH image program. Data were normalized using the respective loading controls.

### Co-immunoprecipitation of HDAC1 and DAXX

The presence of physical interaction between HDAC1 and DAXX was examined in non-transfected HiB5 and HiB5 cells transduced with LVVs carrying non-target RNA or HDAC1 shRNAi. Cell culture dishes were placed on ice, washed three times with cold PBS, scraped out in 1 ml of ice-cold PBS containing phosphatase inhibitors (Active Motif, Carlsbad, CA, USA), transferred to a 1.5 microcentrifuge tube and centrifuged for 3 min at 4 °C at 500* g*. Nuclear protein extraction and co-immunoprecipitation procedures were performed using the Nuclear Complex Co-immunoprecipitation kit (Active Motif) according to the manufacturer's protocol. Nuclear proteins (200 μg) were immunoprecipitated by overnight incubation with a chromatin immunoprecipitation-validated rabbit anti-HDAC1 antibody (2ug; 40967 Active Motif), or rabbit IgG (Jackson Labs, West Grove, PA, USA) used as negative control at 4 °C. Protein–antibody immune complexes were separated using protein G magnetic beads (Active Motif) for 1 h at 4 °C, washed (four times) and re-suspended in 20 μl of 2x Loading Buffer containing DTT (Invitrogen) for western blot analysis. Co-precipitated DAXX was detected using a rabbit anti-DAXX antibody at 1:500 (Santa Cruz).

### Statistical analyses

We used Sigma Plot/Sigma Stat (Systat Software, San Jose CA, USA) to perform statistical analyses and graph results. Sample sizes of experiments were chosen (based on previous publications and literature in the field) that resulted in comparable variation between replicates and that insured detection of effects of 25% difference. Data were analyzed via either Kruskal–Wallis test or one-way analysis of variance followed by Student–Newman–Keuls *post hoc* comparisons. In all tests, *P*<0.05 was taken as statistically significant.

## Results

### Co-localization of HDAC1 and DAXX in HiB5 cells and co-localization of HDAC/GAD67 and DAXX/GAD67 in human hippocampus

Specific antibodies against HDAC1 and DAXX and immunofluorescence detection clearly showed that differentiated HiB5 cells express both HDAC1 and DAXX (Figures 1e and h). Both proteins were located in the nucleus.

In human postmortem hippocampal SO of sector CA3/2, GAD_67_-positive neurons express HDAC1 and DAXX; this can be deduced from the fact that no neurons with either red or green fluorescence alone are present in the overlay (Figures 1i and n).

### Inhibition of HDAC1 with shRNAi results in an upregulation of GAD_67_

**Figure 2:**
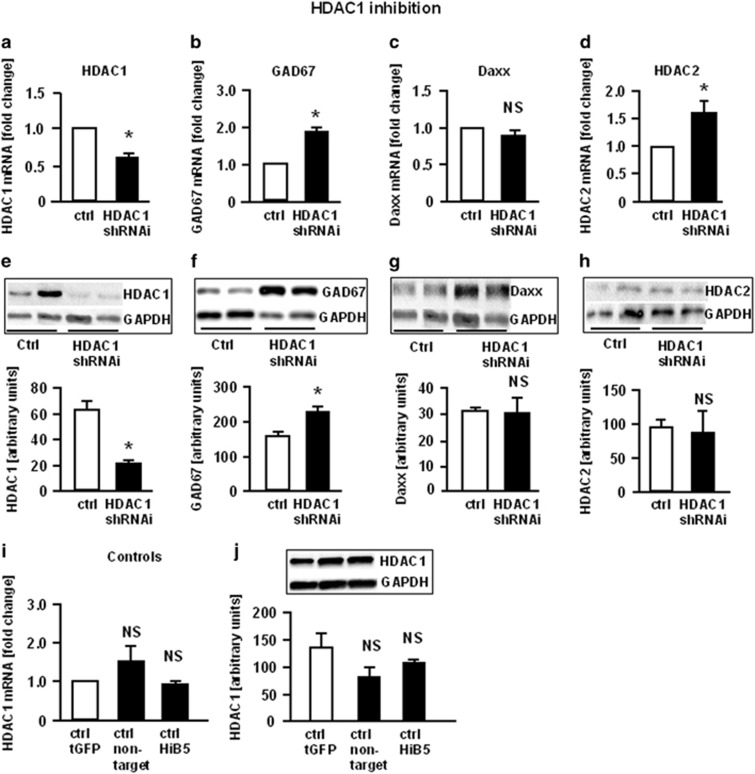
Increase of GAD_67_ mRNA after inhibition of HDAC1 mRNA with shRNAi. Differentiated HiB5 cells were transduced with lentiviral vectors carrying shRNAi for HDAC1. For mRNA analysis, cells were harvested in Trizol, the total RNA extracted and subjected to quantitative RT–PCR. Data are expressed as fold change compared with empty vector (tGFP) control. Statistical significance was evaluated using the Kruskal–Wallis Test, followed by Student–Newman–Keuls *post hoc* test (*P<0.05). Data are expressed as means+s.e.m.; *n*= 6–9 samples per group. (**a**) HDAC1; (**b**) GAD_67_; (**c**) DAXX; (**d**) HDAC2 (*P=0,002) (**i**) comparison of controls: empty vector (tGFP), non-target shRNAi control (will activate RISC and the RNAi pathway but not target any rat genes), differentiated but non-transduced HiB5 cells. For protein analysis, proteins were extracted and western blot with GAPDH as loading control was used for quantification of proteins, which were detected using specific antibodies. Data were analyzed using the public domain NIH Image program (NIH, Bethesda, MD, USA). Statistical significance was evaluated using one-way ANOVA, followed by Student–Newman–Keuls *post hoc* test. Data are expressed as means+s.e.m.; *n*=5–10 samples per group. (**e**) HDAC1 (*P<0,01); (**f**) GAD_67_ (*P<0,05); (**g**) DAXX; (**h**) HDAC2; (**j**) Comparison of controls: tGFP (empty vector), non-target (vectors carrying non-target shRNAi sequence), HiB5 (non-transduced cells); ANOVA, analysis of variance; NS, not significant; RT–PCR, reverse transcription–PCR; shRNAi, short hairpin RNA interference.

To assess the influence of HDAC1 on the expression of the key GABA enzyme, GAD_67_, HiB5 cells were transduced with LV carrying shRNAi for HDAC1. HDAC1 inhibition with shRNAi resulted in a 40% downregulation of HDAC1 mRNA (Figure 2a). This was paralleled by a 60% downregulation of HDAC1 protein (Figure 2e). Inhibition of HDAC1 also resulted in a twofold increase of GAD1 mRNA (Figure 2b) and a 35% increase of GAD_67_ protein (Figure 2f). Expression of DAXX mRNA or -protein was not changed (Figures 2c and g). A comparison of the controls (non-transduced HiB5, empty LV and LV carrying a non-target sequence) showed that the transduction process alone did not influence HDAC1 expression (Figures 2i and j). Since inhibition of HDAC1 often coincides with a compensatory upregulation of HDAC2, we also assessed HDAC2 mRNA and protein expression. HDAC2 mRNA was slightly, but significantly upregulated after HDAC1 inhibition (Figure 2d); however, this did not translate into a corresponding increase in HDAC2 protein (Figure 2h).

### GAD_67_ expression is increased after inhibition of DAXX

**Figure 3:**
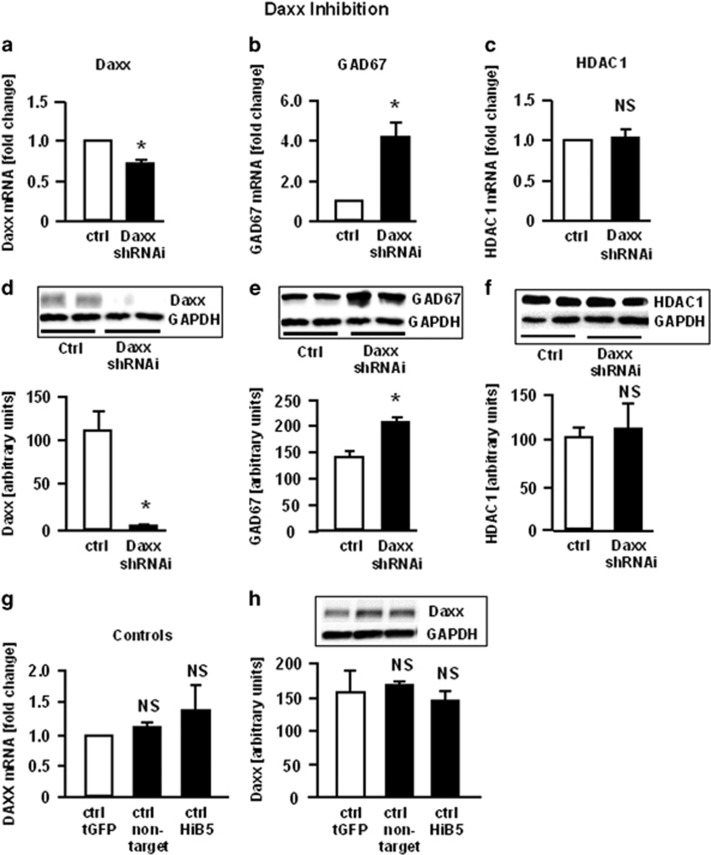
Influence of DAXX shRNAi on GAD_67_ mRNA and protein expression. Differentiated HiB5 cells were transduced with lentiviral vectors carrying shRNAi for DAXX. Quantitative RT–PCR was used to measure changes in mRNA expression. Data are expressed as fold change compared with empty vector (tGFP) control. Statistical significance was evaluated using the Kruskal–Wallis Test, followed by Student–Newman–Keuls *post hoc* test (*P<0.05). Data are expressed as means+s.e.m.; *n*=6–9 samples per group. (**a**) DAXX; (**b**) GAD_67_; (**c**) HDAC1; (**g**) comparison of controls: empty vector (tGFP), vector carrying a non-target shRNAi sequence, differentiated but non-transduced HiB5 cells. Differentiated HiB5 cells were transduced with lentiviral vectors carrying shRNAi for DAXX. Proteins were extracted and western blot with GAPDH as loading control was used for quantification of proteins, which were detected using specific antibodies. Data were analyzed using the public domain NIH Image program. Statistical significance was evaluated using one-way ANOVA, followed by Student–Newman–Keuls *post hoc* test. Data are expressed as means+s.e.m.; *n*=6–8 samples per group. (**d**) DAXX (*P<0,01); (**e**) GAD_67_ (*P<0,05); (**f**) HDAC1; (**h**) comparison of controls: tGFP (empty vector), non-target (vector carrying a non-target shRNAi sequence), HiB5 (non-transduced cells); ANOVA, analysis of variance; NS, not significant; RT–PCR, reverse transcription–PCR; shRNAi, short hairpin RNA interference.

To test whether DAXX plays a role in the regulation of GAD_67_, LV carrying DAXX shRNAi were employed. Transduction of HiB5 cells with DAXX shRNAi LV resulted in a 30% inhibition of DAXX mRNA (Figure 3a); this resulted in a 90% downregulation of DAXX protein (Figure 3d). Inhibition of DAXX increased GAD1 mRNA fourfold (Figure 3b); GAD_67_ protein was increased by about 30% (Figure 3e). Expression of HDAC1 mRNA or -protein was not influenced by DAXX inhibition (Figures 3c and f). Again, controls (empty vector, non-target vector and non-transduced HiB5 cells) did not show any significant difference (Figures 3g and h).

### DAXX is associated with HDAC1 in HiB5 cells

**Figure 4:**
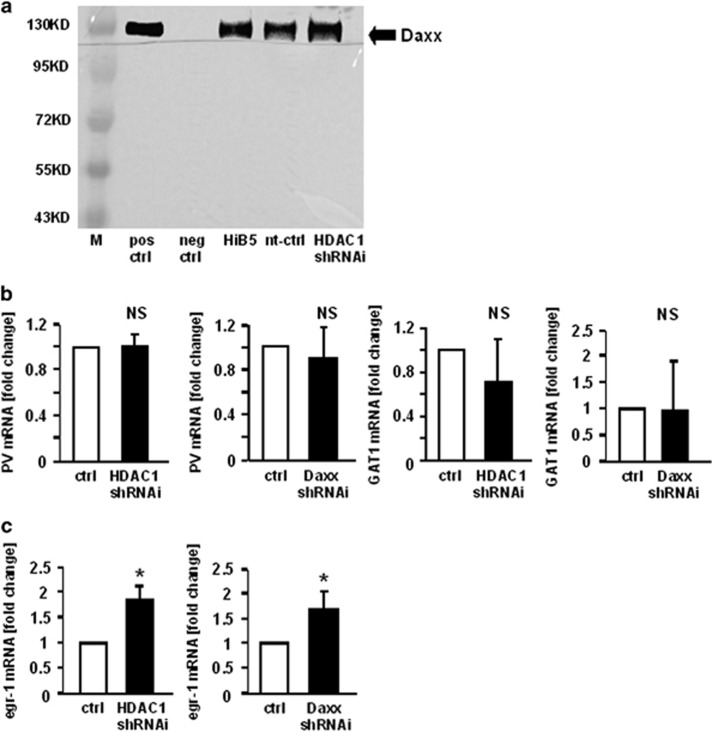
Co-Immunoprecipitation of HDAC1 and DAXX demonstrates physical interaction between the two regulatory factors in HiB5 cells; HDAC1/DAXX do not influence GABA neuron phenotype markers, but regulate egr-1 expression. (**a**) HiB5 cells were differentiated and transduced with a lentiviral vector carrying shRNAi for HDAC1 or a control vector. A specific antibody against HDAC1 was used to co-immunoprecipitate DAXX; visualization was carried out using western blot and a DAXX-specific antibody. HDAC1 shRNAi, HiB5 cells transduced with lentiviral vector carrying shRNAi for HDAC1; HiB5, untreated; M, marker; neg. ctrl, without HDAC1 antibody; nt ctrl, HiB5 transduced with non-target shRNAi sequence carrying control vector; pos ctrl, HiB5 cell extract. (**b** and **c**) To assess PV, GAT1 and Egr-1 regulation, differentiated HiB5 cells were transduced with lentiviral vectors carrying shRNAi for DAXX. Quantitative RT–PCR was used to measure changes in PV, GAT1 (**b**) or egr-1 mRNA (**c**) expression. Data are expressed as fold change compared with empty vector (tGFP) control. Statistical significance was evaluated using the Kruskal–Wallis Test, followed by Student– Newman–Keuls *post hoc* test (*P<0.05). Data are expressed as means+s.e.m.; *n*=5 samples per group; NS, not significant; RT–PCR, reverse transcription–PCR; shRNAi, short hairpin RNA interference.

The possibility of physical interaction between HDAC1 and DAXX in HiB5 cells was assessed using co-immunoprecipitation. Western blot analysis using an anti-DAXX antibody on complexes precipitated with anti-HDAC1 antibody revealed a 120-kDa band corresponding to the molecular size of a DAXX isoform.^13, 23^ There was no difference between untreated differentiated HiB5 cells and samples treated with LVV carrying control non-target RNA or HDAC1 shRNAi (Figure 4a).

### Inhibition of HDAC1/DAXX does not influence GABA neuron phenotype markers

To evaluate whether other GABA neuron phenotype markers besides GAD_67_ are also regulated by HDAC1/DAXX, we exemplary studied GAT1 and PV expression. GAT1, a GABA transporter, which was upregulated during differentiation of HiB5 cells,^19^ was not changed by HDAC1/DAXX inhibition (Figure 4b).

PV is a marker protein for a subpopulation of hippocampal GABAergic neurons instrumental in regulating cortical rhythms underlying cognitive function. Cognitive function is impaired in SZ. While PV is expressed in HiB5 cells, it was not regulated by either HDAC1 or Daxx inhibition (Figure 4b).

### Inhibition of HDAC1 or Daxx increases egr-1 transcription in HiB5 cells

Egr-1 had previously been shown to regulate GAD_67_ expression.^24, 25^ Therefore we tested whether egr-1 expression was influenced by inhibition of HDAC1/DAXX. Quantitative PCR showed that inhibition of either HDAC1 or DAXX increased egr-1 expression (Figure 4c).

## Discussion

Our results demonstrate that the epigenetic factors HDAC1 and DAXX are co-localized with each other in GABA neurons in human hippocampal SO of CA3/2 and have the ability to regulate the expression of the GAD1 gene in hippocampal neurons *in vitro*. The findings reported herein suggest the products of these two genes could play a central role in the normal and abnormal regulation of GABA neuron function in SZ, particularly since they are both significantly upregulated at a locus where GAD_67_ expression is reduced in this disorder.

A prevalence of postmortem abnormalities associated with GABAergic dysfunction in the hippocampus has been found in layer CA3/2 of SO in SZ.^6, 26^ Using gene expression analysis of human postmortem hippocampal tissue followed by a network association analysis, a number of genes were identified that are differently regulated in SZ; some of them had not previously been linked to the disease.^8^ In the specific brain region CA3/2 of the SO, expression of GAD_67_, key GABAergic enzyme responsible for the generation of GABA from glutamate, was decreased. Associated was an increased expression of HDAC1 and DAXX, proteins acting as epigenetic regulators and transcription factors. This distinct expression pattern was limited to CA3/2 of SO; expression of these two regulatory factors was not increased within CA3/2 in the neighboring hippocampal cell layers stratum pyramidale or stratum radiatum, and was not increased in any cell layer of hippocampal sector CA1.^8^ Also, the pattern was specific for SZ; it was not observed in brains from subjects with BD, a disease also associated with GABA dysfunction in the hippocampus. The study of gene CNVs revealed a similar pattern: DAXX CNVs were specifically increased in SO of CA3/2, but not in SO of CA1 and not in hippocampi from BD subjects.^11^ Recently, we showed that methylation changes are enriched in DAXX at specific loci within the hippocampus of patients with SZ.^27^ Although SZ has a significant genetic component, the genetic risk factors are complex and remain elusive. Spatial patterns of expression of risk-associated genes can identify neurons and circuits involved in the disease.^28^ Genes exhibiting CNV are good candidates for research attempting to dissect mechanisms of disease susceptibility,^29^ especially integrated in a context of concordant mRNA regulation. Hence, the specificity of the observations prompted us to examine gene regulation and underlying mechanisms in this region more closely.

There are limits as to what postmortem studies can achieve: first, they are subject to many potential confounds (for example, medication, substance abuse, smoking, perimortem factors), which makes it difficult to distinguish between changes intrinsic to the illness and compensatory or epiphenomenal effects. Second, results are descriptive and correlational.^18^ Therefore, we had established an *in vitro* model of neurons with a GABAergic phenotype.^19^ Basis for this model is the immortalized embryonic day 16 (E16) hippocampal progenitor cell line HiB5. The use of immortalized E16 hippocampal cells, a stage that just precedes the beginning of diversification into neurons and glia in developing hippocampus, ensures that the cultured cells have undergone major developmental stages in an environment that is similar to their *in vivo* counterparts before being kept in culture. A unique combination of growth factors, most prominently BDNF,^21, 22^ directs this differentiation of HiB5 cells towards a GABAergic neuron phenotype with a neuronal morphology (neurites) and GABA neuron markers (GAD_65_, PV, glutamate receptors). Differentiated HiB5 cells show increased expression of GAD_67_ mRNA and protein, and produce GABA and release it on stimulation. Genes which are differentially regulated in the hippocampus in SZ, namely HDAC1 and DAXX, are also expressed in differentiated HiB5 cells.^19^

Using the HiB5 cells with a GABAergic phenotype as an *in vitro* model for postmortem findings in SZ, we demonstrate that changes in the expression of HDAC1 and DAXX result in changes in the expression of GAD_67_. Inhibition of HDAC1 using shRNAi resulted in a concordant increase of GAD1 mRNA and its corresponding protein, suggesting that HDAC1 represses the GAD1 promoter. The effectiveness of inhibition by shRNAi was well within the range achieved in other studies that have employed this approach.^30, 31^ Complete inhibition of HDAC1 was not achieved; but, this would not have been desirable because of the multiple roles of this protein in cell function. HDAC1 inhibits transcription by removing acetyl groups from key histones;^32^ it is not a highly selective agent, as it affects the activity of a number of proteins and contributes to the regulation of cell proliferation and cell death.^33^ One of its principal actions is to serve as a repressor of promoters, including that of the GAD1 gene.^16, 17^ We also tested whether other GABA neuron phenotype markers are regulated by HDAC1/DAXX. Neither the GABA transporter GAT1 nor PV was influenced by inhibition of either protein, suggesting that the observed effects are specific for the key GABAergic enzyme. This is in agreement with the original data from human SZ postmortem hippocampus, where no GABA neuron markers besides GAD appeared to be regulated.

In cooperation with HDAC2, HDAC1 works as a developmental regulator of synapse maturation and function in the brain.^34^ Here, HDAC2 mRNA was upregulated in response to HDAC1 inhibition. This phenomenon has been described before and likely represents a compensatory mechanism which preserves function if an important and ubiquitous regulator like HDAC1 is incapacitated.^35^ However, in our study HDAC2 protein was not changed, which renders compensation of HDAC1 by upregulation of HDAC2 unlikely in this context.

DAXX acts as a co-repressor through an association with HDAC1.^13^ Evidence from co-immunoprecipitation experiments suggest that HDAC1 and DAXX interact physically in GABAergic HiB5 cells. As with HDAC1, DAXX inhibition by shRNAi resulted in an increase of GAD_67_ mRNA and protein. This inversely mirrors the increase of both HDAC1 and DAXX expression associated with decreased GAD_67_ expression in SZ post-mortem hippocampus. Although both HDAC1 and DAXX regulated GAD_67_ expression, they did not influence the expression of each other. HDAC1 inhibition did not change expression of either DAXX mRNA or -protein, nor was HDAC1 affected by inhibition of DAXX. This clearly suggests that HDAC1 and DAXX are not upstream or downstream of one another in a pathway and cooperate to regulate GAD1. In GABAergic HiB5 cells, HDAC1 may be acting through the formation of a complex with the transcription factor DAXX.

The GAD1 gene has a consensus sequence for the transcription factor Egr-1 in its promoter,^36^ and Egr-1 has been shown before to regulate the GAD1 promoter.^24, 25^ Testing the hypothesis that indirect effects might contribute to GAD_67_ regulation by HDAC1/DAXX, we demonstrate that inhibition of these factors resulted in increased egr-1 expression. These findings suggest that an indirect effect, that is, suppression of the transcription factor Egr-1 by HDAC1/DAXX, contributes to reduced GAD_67_ expression in SZ. More detailed promoter studies will be necessary to dissect the exact mechanisms contributing to GAD_67_ regulation by HDAC1/DAXX.

Onset of SZ occurs during early adulthood and is thought to be the consequence of a disturbance of early brain development.^37, 38^ Epigenetic regulation is essential for normal neurodevelopment, and evidence from a number of studies suggests that epigenetic machinery contribute to the etiology of SZ.^39^ The best studied epigenetic mechanisms include DNA/histone methylation, histone acetylation and phosphorylation, changing chromatin structure and allowing the access of transcriptionally active proteins that might activate or repress promoters.^40, 41, 42^ GAD_67_ mRNA and protein are reduced in postmortem hippocampal tissue from SZ patients. This coincides with hypermethylation of histone H3K27, a marker for transcriptional repression, and hypomethylation of H3K4, indicator of transcriptional activation, in the promoter region of the GAD1 gene.^43, 44^ Epigenetic regulators like DNMT1, expressed in GABA interneurons in the adult brain, and histone deacetylase 1 (HDAC1), a DNMT1 binding partner, are upregulated in the brain of SZ patients.^8, 45, 46^ The HDAC1-binding partner DAXX also interacts with chromatin modifiers such as DNA methyl transferases (DNMTs) and CREB-binding protein.^47, 48^

Clinical data support the hypothesis that epigenetic regulation is contributing to GABA neuron dysfunction in SZ. The unspecific HDAC inhibitor valproate jointly acting with the antipsychotic drug clozapine, a combination used clinically in the treatment of SZ, alleviates symptoms of the disease.^49^ Studies on epigenetic regulation of the GAD1 promoter in mice show that clozapine, together with valproate, increases DNA demethylation in a synergistic potentiation.^12, 50^ This, in turn, results in chromatin remodeling that reduces the GABAergic dysfunction in SZ via de-repression of the GAD1 promoter.

**Figure 5:**
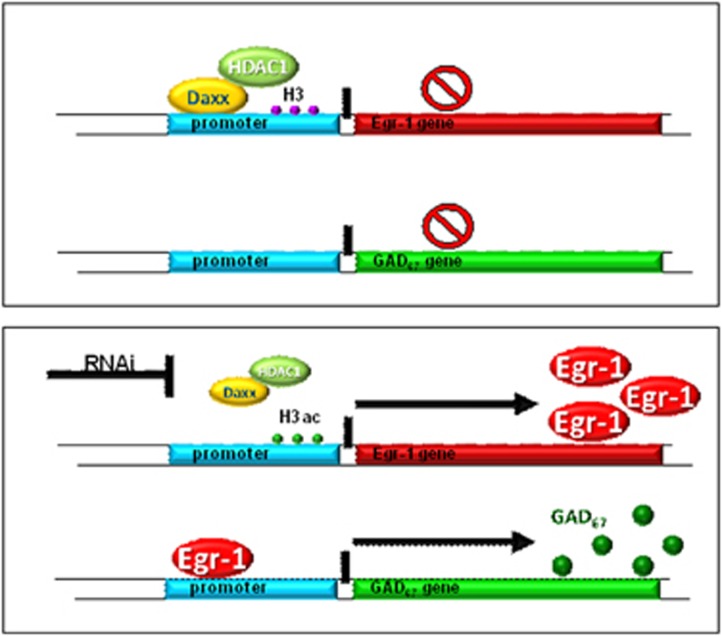
Hypothetical model of GAD_67_ regulation by HDAC1/DAXX. In SZ, HDAC1 and DAXX are increased and form a complex which binds to the egr-1 promoter, inhibiting expression of Egr-1. This results in an indirect inhibition of the GAD_67_ promoter which in turn is dependent on activation by Egr-1. Under normal physiological conditions, or after inhibition of HDAC1 or DAXX, HDAC1/DAXX is decreased in comparison to the situation in SZ, HDAC1/DAXX is no longer associated with the Egr-1 promoter, Egr-1 is expressed and activates the GAD1 promoter. Transcription of the GAD_67_ gene takes place.

In summary, our study has demonstrated that HDAC1 and DAXX regulate the GAD_67_ promoter in a cell-autonomous manner in GABAergic neurons *in vitro,* possibly involving Egr-1, confirming a major role of these epigenetic/transcription factors in the functional differentiation of hippocampal neurons with a GABAergic phenotype. We propose a model (Figure 5) where an HDAC1 and DAXX-containing protein complex binds to the Egr-1 promoter to inhibit the gene's function. In SZ, HDAC1 and DAXX are overexpressed in GABAergic interneurons of SO in CA3, where they induce deacetylation at the Egr-1 promoter and decreased activity of the Egr1 gene. In non-SZ subjects, or after inhibition of HDAC1 or DAXX, the HDAC1/DAXX complex dissociates from the promoter, leading to acetylation of the region and increased transcription of the GAD1 gene. The transcription factor Egr-1 then binds to its consensus sequence in the GAD1 promoter, increasing transcription of GAD67 mRNA.

## Figures and Tables

**Table 1:** Short hairpin RNA interference target sequences

*Gene*	*Target sequence*	*Position*
*HDAC1: XM_576595*		
Target 1	5′-AGCGACGACTACATCAAATTC-3′	232–254
Target 2	5′-ATGGCTATACCATCCATAATG-3′	935–957
Target 3	5′-AGACCCTGACAAACCAATTTC-3′	1230–1252

*DAXX: NM_080891*		
Target 1	5′-AGCTCTGCACTGTCCTTAAAG-3′	385–407
Target 2	5′-AGGTCATAGAGCAGCGAATTC-3′	745–767
Target 3	5′-AGGAGGAGGAAGGAGATAATG-3′	1420–1442
